# Medial Radial Head Dislocation Associated with a Proximal Olecranon Fracture: A Bado Type V?

**DOI:** 10.1155/2014/723756

**Published:** 2014-03-17

**Authors:** Neil Segaren, Hani B. Abdul-Jabar, Nicholas Segaren, Matthew Barry

**Affiliations:** ^1^The Catterall Unit, Royal National Orthopaedic Hospital, Brockley Hill, Stanmore, Middlesex HA7 4LP, UK; ^2^School of Public Health, Harvard University, 677 Huntington Avenue, Boston, MA 02115 6018, USA; ^3^The Royal London Hospital, Whitechapel Road, London E1 1BB, UK

## Abstract

The Monteggia fracture is relatively rare. We present an unreported configuration of a traumatic olecranon fracture with a concomitant medial radial head dislocation in a 3-year-old male. This injury was initially missed and required a subsequent operative intervention. Following surgery, there was evident fracture union, articular congruency, and a full functional recovery. Medial radial head dislocation is not accounted for in the Bado (1967) classification of the Monteggia lesion and hence we propose the addition of a Bado V category.

## 1. Introduction

The Monteggia fracture is relatively rare in the paediatric population and has been classified by Bado [1] into four variants. We present the case of a three-year-old boy who sustained an olecranon fracture with a concomitant medial dislocation of the radial head. We propose the addition of a 5th variant to the Bado classification to encompass medial and anteromedial radial head dislocations.

## 2. Case Report

The patient's guardian gave informed consent prior to the child being included into the study. This study was authorized by the local ethical committee and was performed in accordance with the Ethical standards of the 1964 Declaration of Helsinki as revised in 2000.

**Figure 1:**
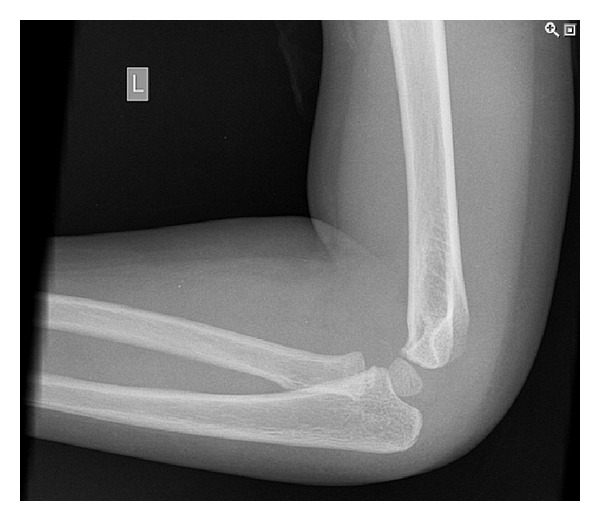
Lateral radiograph of three-year-old boy showing olecranon fracture.

**Figure 2:**
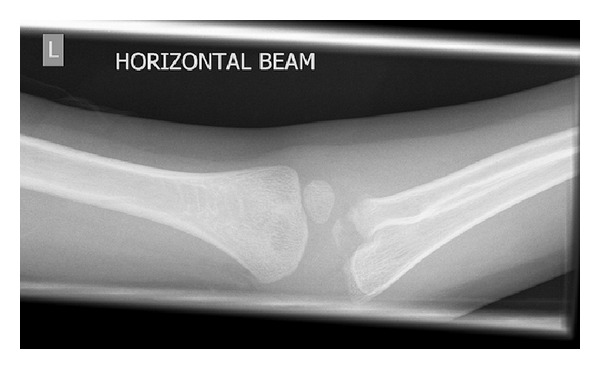
Anterioposterior radiograph: note the bony fragment and the medial radial head dislocation.

**Figure 3:**
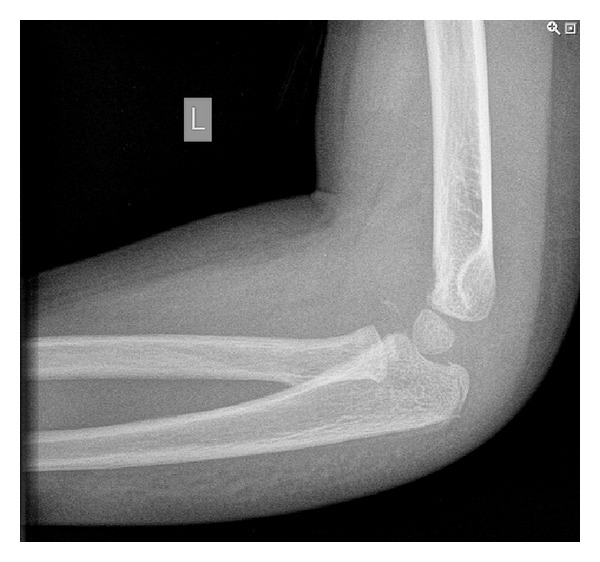
Repeat lateral radiograph of the same elbow twelve days later. The olecranon fracture is more defined.

**Figure 4:**
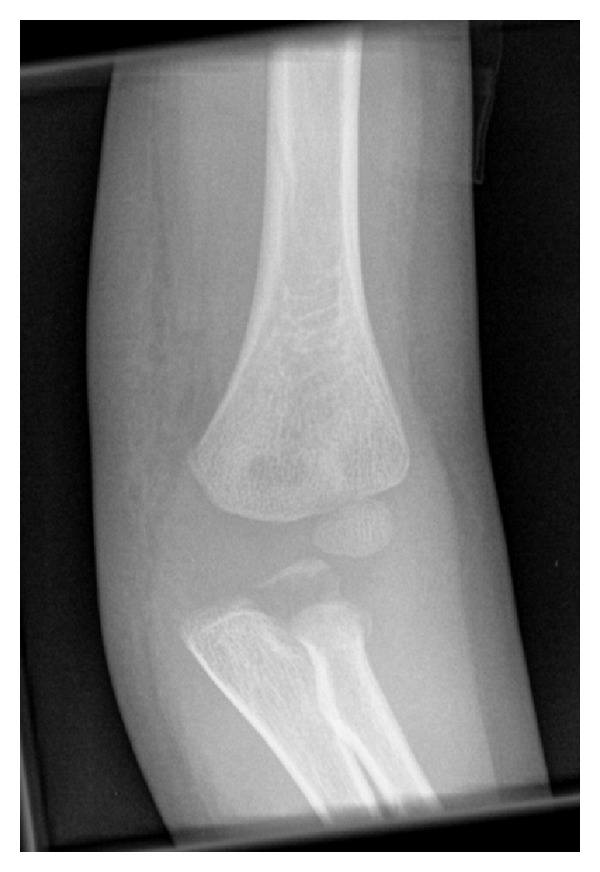
Anterioposterior radiograph of the same elbow twelve days after injury. Bony fragment still visible.

A three-year-old boy fell whilst running in a playground. He presented to the emergency department at his local hospital and was complaining of a painful elbow. On examination, his elbow was noted to be extremely swollen and has adapted a flexed posture with maximal apprehension and reluctance at any attempt to mobilise its joints in all directions. Plain radiographs were obtained (Figures 1 and 2) which confirmed an olecranon fracture in the presence of a radial head dislocation. The radial head dislocation was not initially recognised by the assessing casualty doctor. The elbow was immobilised in an above elbow back slab and the patient was discharged home with a planned follow-up in fracture clinic where he was seen by an orthopaedic registrar 13 days after the injury. Repeat radiographs were obtained (Figures 3 and 4) and the medial radial head dislocation was confirmed.

Due to the nature of this unusual presentation; the child was referred to the regional tertiary paediatric orthopaedic centre for further assessment in view of a possible need for open reduction and operative intervention. Fifteen days after injury, the child was taken to the operating theatre; initial examination under anaesthesia revealed flexion-extension arc from 0 to 130 degrees and 5 degrees each of supination and pronation.

A direct posterior approach was utilised to openly expose the olecranon fracture which showed signs of early callus deposition. The olecranon fracture was mobilised fully beyond its assumptive initial fracture position; allowing adequate visualisation of the radial head, which had surprisingly dislocated medially. The radial head was reduced under direct vision, the ulnar fracture was then anatomically reduced and stabilised with two crossed Kirschner wires. Examination following articular congruency and fracture fixation demonstrated a full range of anatomical supination-pronation arc.

**Figure 5:**
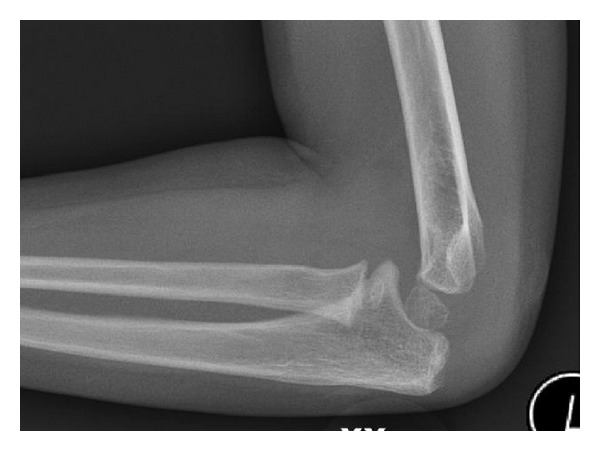
Lateral radiograph four months postoperatively. Note the union of the olecranon fracture and the satisfactory position of the radiocapitellar joint.

**Figure 6:**
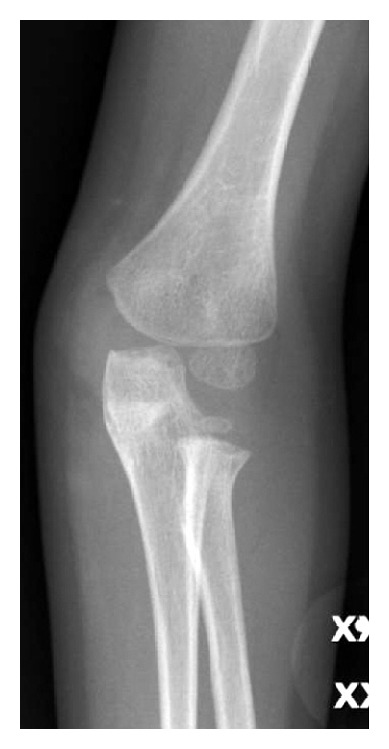
Anteroposterior radiograph four months postoperatively. Note the union of the olecranon fracture and the satisfactory position of the radiocapitellar joint.

Postoperatively, the arm was immobilised for four weeks in an above elbow plaster of Paris. The wires were removed following plaster disposal and the patient was encouraged to actively mobilise his elbow as comfort allowed. At his last follow-up 8 months after open reduction and internal fixation, the child had a pain-free elbow with a full arc range of movement. Radiographs (Figures 5 and 6) demonstrated full union of the olecranon fracture and elbow joint articular congruency.

## 3. Discussion

Monteggia fractures are uncommon and account for about 0.7% of elbow fractures and dislocations [2] and 7% of fractures of the radius and ulna [3]. Bado [1] classified the Monteggia lesion into four types (Table 1), depending on the direction of the radial head dislocation. Traumatic medial dislocation of the radial head associated with a proximal olecranon fracture is a rare injury and to our knowledge has not been reported within the scientific literature to date. Anteromedial dislocation of the radial head has been described in low energy trauma in association with an undisplaced olecranon fracture [4] and with [5] or without [6] radial head fracture, and in isolation as a consequence of birth injury [7].

Due to the unique fracture configuration, the radial head dislocation was not identified for 15 days. The senior author felt that an open reduction offered the greatest chance of an optimal outcome.

In an isolated anteromedial radial head dislocation, closed reduction alone is often unsuccessful due to soft tissue interposition from the annular ligament [8], biceps tendon [5, 6], median nerve [9], radial nerve [10], or joint capsule [4, 11]. The direct posterior approach used in this case provided a safe operative window allowing full visualisation and subsequent reduction of the dislocated radial head and anatomical fixation of the associated olecranon fracture.

In summary, we report a traumatic medial radial head dislocation associated with a displaced olecranon fracture. We do not believe that this pattern has been reported in the literature although four cases of anteromedial dislocation have been reported [4–7]. Bado [1] stated that any dislocation (of the radial head) found with an ulna fracture constitutes the anatomical-clinical picture of the lesion discussed by Monteggia. The current classification of the Monteggia lesion does not account for medial or anteromedial radial head dislocation; hence the authors propose the addition of a Bado type V (Table 1).

## Figures and Tables

**Table 1:** Bado [1] classification of the Monteggia lesion with the proposed type V category.

Bado Type	Description
Type I (60%)	Anterior dislocation of the radial head. Fracture of the ulna with anterior angulation.
Type II (15%)	Posterior or postero-lateral dislocation of the radial head. Fracture of the ulna with posterior angulation.
Type III (20%)	Lateral or antero-lateral dislocation of the radial head. Fracture of ulna metaphysis.
Type IV (<5%)	Anterior dislocation of the radial head with fracture of the proximal third of the radius and fracture of ulna at same level.
Type V (proposed)	Medial or antero-medial dislocation of the radial head with fracture of the proximal ulna.
